# Interactions of Nanoparticles and Biosystems: Microenvironment of Nanoparticles and Biomolecules in Nanomedicine

**DOI:** 10.3390/nano9101365

**Published:** 2019-09-24

**Authors:** Carlota Auría-Soro, Tabata Nesma, Pablo Juanes-Velasco, Alicia Landeira-Viñuela, Helena Fidalgo-Gomez, Vanessa Acebes-Fernandez, Rafael Gongora, María Jesus Almendral Parra, Raúl Manzano-Roman, Manuel Fuentes

**Affiliations:** 1 Department of Medicine and General Cytometry Service-Nucleus, Cancer Research Centre (IBMCC/CSIC/USAL/IBSAL), 37007 Salamanca, Spain; idu007523@usal.es (C.A.-S.); tabata.nesma@gmail.com (T.N.); pablojuanesvelasco@usal.es (P.J.-V.); alavi29@usal.es (A.L.-V.); hfidalgogomez@gmail.com (H.F.-G.); vanessaacebes@usal.es (V.A.-F.); rgongora@usal.es (R.G.); 2 Department of Analytical Chemistry, Nutrition and Food Science, Faculty of Chemistry, University of Salamanca, 37008 Salamanca, Spain; almendral@usal.es; 3 Proteomics Unit. Cancer Research Centre (IBMCC/CSIC/USAL/IBSAL), 37007 Salamanca, Spain

**Keywords:** nanoparticles, interactions, protein corona, nanomedicine, biomolecules

## Abstract

Nanotechnology is a multidisciplinary science covering matters involving the nanoscale level that is being developed for a great variety of applications. Nanomedicine is one of these attractive and challenging uses focused on the employment of nanomaterials in medical applications such as drug delivery. However, handling these nanometric systems require defining specific parameters to establish the possible advantages and disadvantages in specific applications. This review presents the fundamental factors of nanoparticles and its microenvironment that must be considered to make an appropriate design for medical applications, mainly: (i) Interactions between nanoparticles and their biological environment, (ii) the interaction mechanisms, (iii) and the physicochemical properties of nanoparticles. On the other hand, the repercussions of the control, alter and modify these parameters in the biomedical applications. Additionally, we briefly report the implications of nanoparticles in nanomedicine and precision medicine, and provide perspectives in immunotherapy, which is opening novel applications as immune-oncology.

## 1. Introduction

One definition of nanotechnology comes from the statement by the US National Science and Technology Council [1], which states: “The essence of nanotechnology is the ability to work at the molecular level, atom by atom, to create large structures with fundamentally new molecular organization. The aim is to exploit these properties by gaining control of structures and devices at atomic, molecular, and supramolecular levels and to learn to efficiently manufacture and use these devices.” Other authors describe nanotechnology as the combinatorial study and integration of scientific technological advances and medical engineering at the nanoscale level [2,3]. All these definitions cover the design and manipulation of nanomaterials. Therefore, nanomaterials, which are one dimension smaller than 100 nanometers, enhance physical, chemical, and biological properties of the original material [4].

Over the last few years, nanotechnology and related disciplines have undergone an exponential growth in applications such as nanomedicine, energy and electronics, and the environment and materials because of the unique properties of nanomaterials [5]. Nanomedicine involves the development of nanoparticles (NPs), among other nanocomponents and devices. For the molecular diagnostics, nanomedicine includes treatment and prevention of human diseases thanks to their compatibility with biomolecules [6].

Currently, NPs have an impact and show an increasing presence in many scientific designs and developments [7]. These have a number of disadvantages, such as the cytotoxic effects in living organisms, which may limit their use within the clinical setting [8]. However, important advantages, which make them an ideal approach for biomedical applications, such as their intrinsic ability to enter the human body through inhalation, the skin and digestive routes depending on their physicochemical properties potentially accesses vital organs through the blood flow [9]. However, some key factors must be taken into consideration during the bio-nano-interface construction: (i) The interaction of nanoparticles with their ecosystem, mainly with other nanomaterials and biomolecules. Some studies show the possibility of using AgNPs as antibacterial agents thanks to high toxicity against human pathogenic bacteria. In this sense, Singh T. et al. have demonstrated the use of endophytic fungi Alternaria sp. to synthetize AgNPs [10]. (ii) Their physicochemical properties achieve a suitable design such as particle size, shape, dispersity, surface charge, and protein corona effects. Protein corona is a complex plasma proteins layer around the NPs that takes place after systemic administration, when nanoparticles are exposed to physiological proximal fluids, which is mostly blood.

The adsorption of dozens of proteins with varying identities and quantities on the NPs can modify their physicochemical identity, cellular uptake, targeting, circulation lifetime in the blood, and influence the physiological response and toxicity [11]. In this sense, a number of molecules can be used to maintain the integrity and stability of NPs in biological fluids [12]. Dutta P.H. et al. synthesized and characterized two types of NPs, AgNPs, and AuNPs, in order to design an antimalarial nanomaterial. For it, they optimized the size, shape, and surface morphology of the bio-synthesized NPs and showed that AgNPs had insignificant and lower cytotoxicity against several human cancer cell lines than AuNPs. [13]. (iii) As well as the interacting bio-compounds (biomolecules, cells, proximal fluids) that favor a physical, chemical, and mechanical relevant process [1,5]. Mirkin showed that siRNA-based gold nanoparticles inhibit its enzymatic degradation and facilitates its uptake by Hela cells [14].

Despite their potential advantages and promising applications, NP entrance in a physiological environment may be problematic due to different intrinsic NP characteristics. These particles may appear embedded in human proximal fluids, inside cells and culture media among others. Thus, there are multiple conditions and a huge variety of biomolecules potentially interacting with the NPs. This previous knowledge is important for predicting their impact [15]. Because of these inherent interactions, the NPs might have a heterogeneous morphology, which is also correlated with the resulting immuno-biocompatibility and safety of these nanomaterials.

In this case, this mini-review focused on the global interactions of NPs and biomolecules in biological environments, which play a critical role in biomedicine applications.

## 2. Nanoparticle-Cell Dynamics

**Figure 1:**
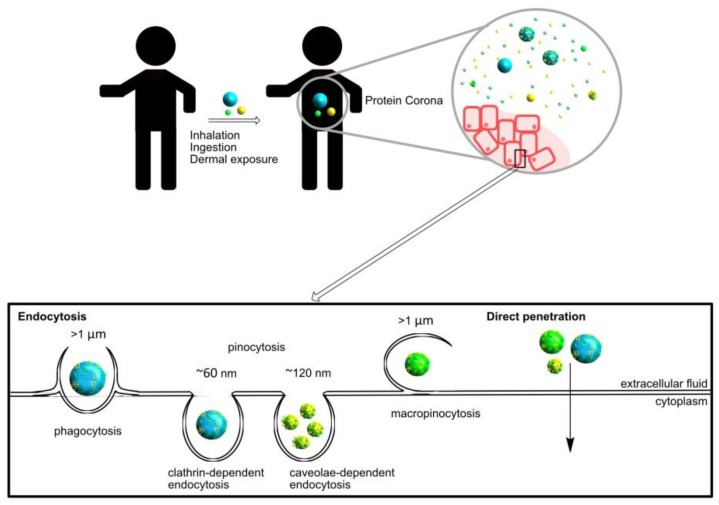
Schematic representation of different ways to enter nanoparticles (NPs) in the human body and inside cells.

In general, NPs enter the cells through different internalization mechanisms (Figure 1), accumulate in targeted organs, and are later eliminated.

Their small size allows them to enter the human body by inhalation, ingestion, or through the skin. Once in the extracellular fluid, they are conjugated with biomolecules presented in the media, which allows them to internalize in the cells.

### 2.1. Cellular Internalization

Nanomedicines based on NPs must cross the cell membrane by different mechanisms (phagocytosis, micropinocytosis, clathrin-dependent endocytosis, caveolae-dependent endocytosis, or by direct penetration) to produce an effect inside the cells [16] because, usually, cell membranes are impermeable to NP diffusion. Moreover, non-specific internalization mechanisms may induce toxicity. Since a lack of biocompatibility is desirable, it becomes a suitable mechanism for internalization [17].

As expected, the NP size directly affects the internalization process. In fact, NPs in the range of 10–100 nm achieve higher cellular uptake and, on the other hand, small ones imply a higher energy cost to the cells [18]. Usually, NPs larger than 100 nm are internalized by specialized phagocytic cells (such as macrophages, dendritic cells) which allows targeted design.

Furthermore, the optimal size for internalization inside the cells is strongly linked to the NPs surface chemistry. In general, Van der Waals or electrostatic forces are critical in the NP interactions with biomolecules and cells. In fact, several studies show correlation between zeta potential and endocytosis/exocytosis mechanisms [19]. Then, specific cellular internalization could be targeted to favor specific interactions (i.e., employing affinity ligands) as opposed to nonspecific interactions (i.e., hydrophobic). In this sense, antibody-coated NPs present an internalization potential in targeted cells four to eight folds higher than positively or negatively charged NPs without the affinity component [20]. Besides the use of antibodies for targeting delivery, non-specific interactions through chemical moieties are always present and influence target affinities, which must be always taken into consideration.

Protein adsorption also depends on the NP shape and, consequently, affects the cellular uptake. It seems that spherical and highly homogeneous NP conjugates have better cellular uptake than amorphous and non-geometrically symmetric nanoconjugates [21]. Moreover, several authors claim that shape could be employed to prevent non-specific cellular internalization in the targeted cells [19].

### 2.2. Tumor Accumulation

NPs accumulate preferentially in tumor tissues in comparison with the normal ones [22]. This is mainly because the vessels around the tumoral tissue have a higher permeability (than the normal vessels) and tumors have impaired lymphatic drainage, which leads to retention of the permeated NPs. This is an effect called enhanced permeation and retention (EPR) [23,24,25].

Tumors are densely packed with cells and the extracellular matrix. Thus, NP size plays an important role in the diffusion and accumulation inside the tumor. The accumulation within the tumors could be modulated by the NP physical dimensions and surface chemistry. In general, diffusion and NP size are inversely correlated [26]. Small size NPs can diffuse freely across tumoral tissue and present a widespread distribution within normal tissues. However, small NPs can easily and quickly clear out. Size is important for other purposes such as when NPs are applied as imaging agents helping to distinguish normal and pathological tissues because they appear only on the tumor periphery thanks to their bigger size.

As previously discussed, the biomolecules adsorption onto the NP surface is directly related with their opsonization and clearance capacity. Therefore, it is related with the blood concentration along with time.

### 2.3. Elimination

In general, NPs are eliminated from the human body by renal and hepatobiliary routes and need to be done for clinical approval in a reasonable timeframe. Then, drug conjugated NPs must be designed to avoid quick clearance and long period of body maintenance.

As is expected, surface chemistry, shape, and NP size influence elimination. For example, surface chemistry is quite critical in the clearance efficiency -even for small NPs- and polyethylene glycol (PEG) coating promotes more efficient hepatobiliary clearance [27]. Another point is the NP size. The hydrodynamic NP size has a strong influence on the renal clearance, where the glomerular pores are a physical barrier [23,28].

## 3. Nanoparticle Interactions

**Figure 2:**
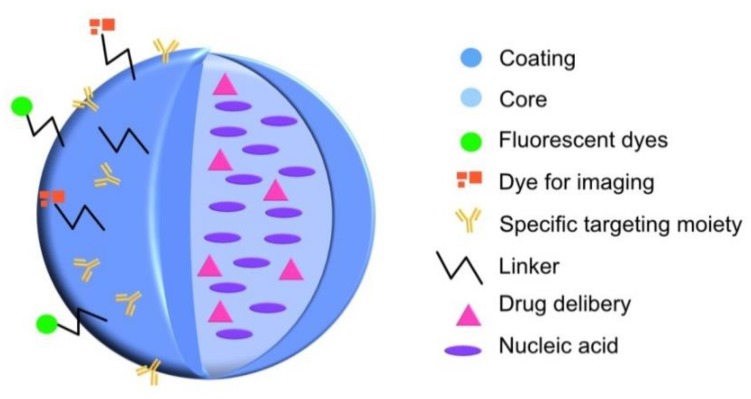
Schematic representation of multi-functional nanoparticles.

Although NPs in biological systems are surrounded by large quantities of biomolecules, depending on the different factors that characterize the biological environment. The NP promotes multiple and different interactions. Multifunctional NPs as nanomedicines (see Figure 2) are embedded in human proximal fluids, inside cells, and inside culture media among others [29]. This implies a huge variety of different microenvironments with additional challenges for the design and development of NPs suitable to be functional in all kinds of conditions. However, depending on the medium conditions like pH [30], ionic strength, oxygen levels, organic matter, etc., NPs present different forms or stages, such as ionized particles [31], which form aggregates or combine into complex aggregates or even interact with other nanomaterials [32]. This is especially relevant because it may be the origin of a heterogeneous morphology, which might be correlated with a lack of stability and immuno-biocompatibility of these nanomaterials [33].

NP aggregation and agglomeration have been recognized to affect cellular uptake and even induce potential toxicity based on the nanoparticle composition and the cell type [34]. Aggregation and agglomeration effects are often used in nanotechnology, but both terms are commonly mistaken. Aggregation indicates strongly bonded or fused particles and it occurs when the Van der Waals attractive forces between particles are greater than the electrostatic repulsive forces produced by the nanostructure surface [34,35]. On the other hand, agglomeration indicates more weakly bonded particles and it does not require a definite pattern, shape, and size [35]. Pellegrino F. et at. studied the agglomeration and aggregation influence on the optical properties of TiO_2_ NPs demonstrating that this effect can lead to an incorrect assessment of the photoactivity [36]. Zook M.J. et al. [37] developed a bottom-up-based method to produce controllable, reproducible, and stable NP agglomerates in an aqueous medium. They used this method to show how silver NP agglomeration affects hemolytic activity.

The main factors that will determine the type of interactions between NPs are: the complementarity between nanomaterials and their distance and geometry [38]. In addition, it is also essential to know what the main interactions drivers are in an NP assembly. For example, Van der Waals forces form nanocrystal superlattice membranes, electrostatic interactions obtain colloidal dimers, and magnetic interactions where iron oxide NPs coated with azobenzene-terminated catechol ligands self-assemble by UV-light-induced, or even molecular force [38].

An example that demonstrates the importance of the complementarity between the materials and the influence of the forces used in such an interaction is one discussed by Pileni and co-workers. They stress the difference of using octanoic and dodecanoic acids as organic ligands in magnemite NPs in the absence (only with dipolar forces between the magnetic nanoparticles) and the presence of Van der Waals interactions, when the distance is small [39,40].

**Figure 3:**
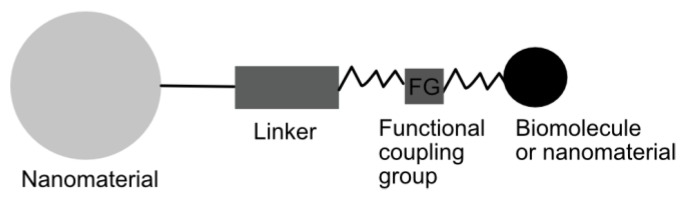
Schematic representation of the strategy to couple nanoparticles and biomolecules or other nanoparticles.

On the other hand, an interaction between molecules on surfaces is highly dependent on surface functionalization (Figure 3). This implies the presence of reactive chemical moieties on the surface being homo-functional or hetero-functional depending on whether there is only one chemical group on the surface or whether different chemical reactive groups co-exist [41].

Due to their composition and structure, the surface might not allow different types of interactions. Thus, for example, circulatory cells are covered by a lipid bilayer with proteins and polysaccharides that, depending on the NP exposed groups, will favor one type of interaction mechanism [42]. Another example includes the proteins affected by their molecular weight, charge (greater adsorption near the isoelectric pH), or its stability that influences the number of binding points [43]. A soft protein layer has a low structural stability and a greater number of active centers to interact with, besides other influencing physicochemical factors on the surface (i.e., humectability). The hydrophobicity/hydrophilic surface ratio influences protein reactivity and/or its adsorption properties. Another remarkable feature is the size, including those with a size comparable to that of the NP, which will be more easily adsorbed.

Lastly, it is not only necessary to consider the concentration or size of NPs, but also the species and quantity of resulting products from chemical interactions between NPs.

### 3.1. Interaction Mechanisms Between Nanoparticles and Biomolecules

There is a wide-open variety of biomolecules, which could interact directly onto the NPs surface or through other biomolecules coating the NPs surface (Figure 2). These layers of coating biomolecules are directly related with the type of organism, biological fluid, cells, etc., among the physicochemical conditions of the media and NP surface, nature, and structure of biomolecules.

According to the literature, the most relevant interacting biomolecules to the NP surfaces are proteins and nucleic acids [44]. Proteins have many different binding sites (as amino acidic key structures and/or post-translational modifications) through specific or non-specific adsorption [43,45]. In addition, the proteins are critical on the immune-biocompatibility of the nanomaterials. Nucleic acids have many different applications as a consequence of its physicochemical stability, mechanical rigidity, easy accessibility, and its high specificity of base pairing, which results in a suitable receptor for molecular nano-construction [46].

Regarding interactions with human biomolecules, two factors must be considered in the description of the interaction [23]. The first one is that NPs in biological systems are surrounded by multiple potentially interacting biomolecules that may modify and saturate their surface. Therefore, custom modified NPs are the ones that may interact specifically with the biomolecules of interest later on. The second factor is NP entering pathways into the human body. This depends on the way it can influence the force of the interaction. For example, NPs entering by inhalation strongly interact with the pulmonary system (proteins and phospholipids).

Two immobilization mechanisms have been studied through an interaction with different types of biomolecules [45]: by simple absorption or by chemical linkages. The immobilization of enzymes on NPs through adsorption is a very useful method because it takes place through non-covalent forces (hydrogen bonding, ionic interactions, and Van der Waal forces), mainly through negatively charged phosphate groups and hydrophobic moieties not disturbing the initial structure of the enzyme or its active site. Immobilization through chemical linkages may lead to the immobilization of biomolecules on a biocompatible matrix, such as within phospholipid bilayers, not interacting with the native structure of the biomolecule and altering its biological activity.

We also find two other types of interaction mechanisms with cells: ligand-receptor interaction and chemical conjugation [47]. An example of the first interaction method is the NP surface functionalization with a receptor, such as streptavidin-biotin. Its non-covalent interaction results in a greater bond strength, which provides resistance to pH, temperature variations, and denaturants. In addition, they have a greater binding affinity to cells. Chemical conjugation simply consists of the coupling of functional groups (such as thiol groups) to the NP surface, which favors subsequent binding to the cell and, in turn, reduces the toxicity of this interaction. A disadvantage of this method is that, in terms of biomedical applications, the covalent binding of the drug to the NP restricts its efficient release, which limits its effectiveness.

### 3.2. Nanoparticle Design: Influence on Interaction Mechanisms

NPs undergo different changes in a concrete environment such as the generation of a coating protein corona once plasma proteins are adsorbed on its surface. Therefore, it is necessary to study the NP states and characteristics prior to interaction assays [48].

Many NP-based investigations focus on issues affecting NP characteristics and, subsequently, their impact on cellular internalization and biodistribution. Centi J. et al. [49] and Tatini J. et al. [50] talk about the interest of gold nanorods (GNRs) in the biomedical field. GNRs are gold NPs that are elongated along one direction with characteristic optical properties, which depend on the particle size and shape [51]. They are attractive in biomedical optics because of their special and intense absorption band near infrared light (650–1000 nm). Other important features of GNRs include their coating, which are crucial for their biological applications, i.e., conjugation with PEG. In addition, their shape and size are critical for modulating cellular penetration, intracellular localization, and bio-distribution. GNRs may become coated with, which may modify their conformation and cause a loss of their biological activity. Bovine Serum Albumin has been chosen as a protein target to investigate NPs coating with polyethylene glycol (NP-PEG) exposition to biological fluids because it is the most abundant protein in the blood and can transport metal compounds. The Tatini J. et al article proposes CA-125 as the molecular target cancer antigen to model “in vitro” some of the most critical issues that arise from the interactions between GNRs and the bloodstream using an analytical approach.

**Figure 4:**
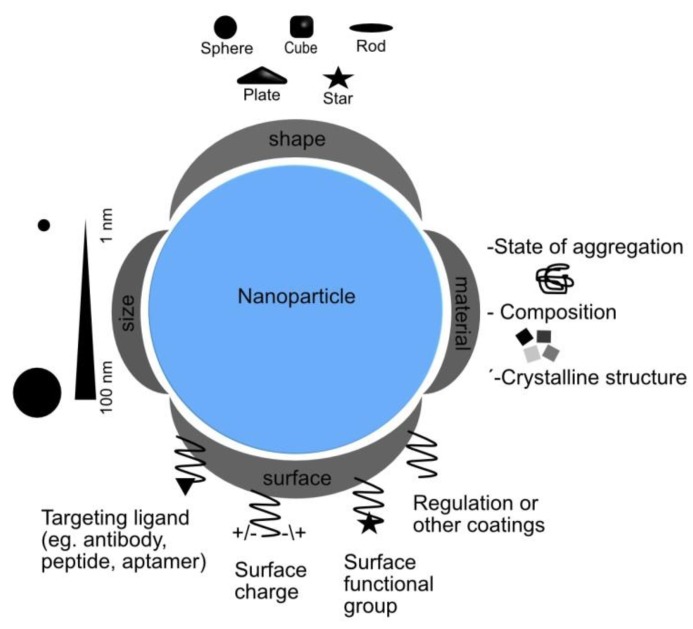
Schematic illustration of the main physicochemical properties of nanoparticles governing interaction mechanisms in biological systems.

The physicochemical properties of NPs (Figure 4, some already commented) represent their identity and influence on the synthetic moieties incorporated [52] among all including size, shape, surface, coating and morphology, surface charge, solubility, chemical composition, crystalline structure, and, lastly, the agglomeration status. These properties will also play a characteristic role in relevant mechanisms such as cellular biocompatibility studies.

#### 3.2.1. Size

Size plays an important role in interactions with the biological system and many biological NP-related mechanisms such as cellular uptake and particle processing efficiency in the endocytic path depending on it [53]. Additionally, the ion release rate, the smaller size, the faster release rate, and the interactions with cell membranes [54]. In general, there is a size-dependent NP toxicity and, therefore, their ability to enter in the human system. As the particle size decreases, the surface/volume ratio increases. Therefore, their contacting surface will increase, which makes penetration into the body easier and increases their toxic effect [54]. NP sizes less than 50 nm through intravenous injection connect to all tissues faster and exert stronger toxic effects [55].

The NP size indicates their “in vivo” distribution, or pharmaceutical behavior [56], and their most direct impact on physiological activity. NP sizes larger than 1 µm cannot easily enter the cell, but they interact with proteins absorbed in the cells. NP sizes greater than 6 nm cannot be excreted by the kindness and accumulate in specific organs [57]. For example, cadmium selenide quantum dots contact stays in the tissue, which causes hepatotoxicity [58].

Sonavane et al. carried out studies on the bio-distribution and bioaccumulation in the blood of gold nanoparticle (AuNP) of different sizes. They observed that smaller ones stayed longer in the bloodstream and accumulated to a greater extent in all organs [59].

#### 3.2.2. Shape

Shape is a physicochemical property that influences the toxicity of materials [60]. NPs have different shapes and structures such as tubes, fibers, spheres, and planes. Therefore, it may also influence their endocytosis process, internalization, bio-distribution, and elimination. For example, spherical nanoparticles of similar size have been found to be easier and faster internalized by endocytosis than rod-shaped nanoparticles, which is explained by a greater membrane wrapping time required for the elongated particles. In addition, the spherical ones are relatively less toxic [21].

#### 3.2.3. Surface Modification

NP-cell interactions and solubility depend on the nature of the NP surface [61]. NP surface coating alteration can modify their magnetic, electrical, chemical, and optical properties, which affects their cytotoxic properties by influencing pharmacokinetics, distribution, accumulation, and toxicity [62].

Surface charges determine the response of the organism to changes in NP shape and size in the form of cellular accumulation, called colloidal behavior [63]. The effect of surface chemistry on NPs affects absorption [64], colloidal behavior, plasma protein binding [65], and crossing the blood-brain barrier [66]. The NP cytotoxicity increased with an increase in surface charge [67]. This suggests that higher positive charges get greater cell electrostatic interactions and, consequently, greater endocytic uptake. However, the uptake of positively charged NPs may produce higher toxicity than negatively charged [68]. NPs with a positively charged surface tended to accumulate more in tumors than negatively charged ones most likely because positively charged density can be more easily separated in the interstitial space and, therefore, internalized by tumor cells [56].

Surface chemical modification is an important strategy utilized in biomedical applications to decreases toxicity, increase stability, and to control and modulate cellular internalization [69]. Surface functionalization is predominantly comprised by polyethylene glycol (PEG), the negative carboxyl group, and neutral groups like hydroxyl group, and amine groups [67]. For example, the NP surface can be functionalized by proper polymers such as PEG to reduce non-specific binding and to get specific binding to cell receptors [70].

Hydrophobicity is another key factor that also affects pharmacokinetics and bio-distribution [70]. NPs with a 2more hydrophobic surface tend to absorb plasma proteins, which reduces the time spent in the bloodstream [71]. A computer molecular simulation study revealed that the surface membrane uptake of hydrophobic C60 agglomerates is thermodynamically favored than semi-hydrophilic ones because of the interior membrane hydrophobicity space in cells [72].

#### 3.2.4. Chemical Composition

NPs chemistry is another fundamental factor contributing to cell interactions. Regarding particle chemistry, Griffitt et al. [73] observed different toxicity in zebrafish, daphnids, and algae species for silver and copper NPs with respect to titanium oxide, which resulted in no toxicity problems.

In addition to these characteristic properties of NPs, their state of aggregation must also be taken into account. Aggregation depends on the surface load, material type, and size, among other factors. It has been shown that higher NP concentrations result in higher aggregation and, consequently, lower toxicity [74]. Accordingly, macrophages remove large particles more efficiently and easily than small ones, which evade this defense mechanism more easily [75].

#### 3.2.5. Protein Corona

**Figure 5:**
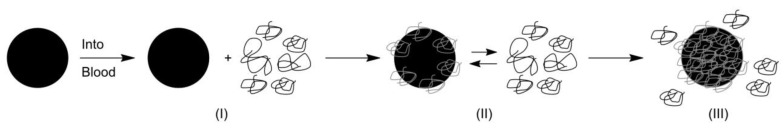
Schematic protein corona formation. First, the introduction of a nanoparticle to fluid/medium enriched in protein content takes place (I). Then, the nanoparticle is coated with proteins, which are abundant and highly mobile (II). Lastly, the protein species are exchanged over time, which results in hard corona of strongly bound proteins (III).

Since NPs are injected into the bloodstream, they are exposed to a large amount of biomolecules that form a corona around them [76] (Figure 5). Protein corona is mainly composed of proteins with different affinity interactions: immunoglobulin G, serum albumin, fibrinogen, clusterin, and apolipoproteins [77]. Therefore, NPs experiment changes in their physicochemical properties and their biological identity once the protein corona is formed. Therefore, in order to know the possible adverse effects of the physicochemical, kinetic, dynamic, and thermodynamic interactions of NPs, the characterization of these NP-protein interactions has become one of the main challenges of nanomedicine.

When NPs are incubated in a biological medium, a competitive dynamic process (between soluble biomolecules and surface) take place to form the protein corona. This process is based on the affinity adsorption of proteins on NP surfaces and on protein-protein interactions. According to the Vroman effect [78], the first are bound to NP surface proteins with a high concentration and low affinity and then are gradually replaced by higher affinity proteins present in low concentrations. The protein corona is classified into hard and soft depending on the duration of protein exchanges. Hard corona is a bound layer of proteins with high affinity and long exchange time. Proteins of the hard corona form the closest layer to the NP surface, so they are susceptible to thermodynamically favorable conformational changes (irreversible) depending on the chemistry functionalization, the hydrophobicity or hydrophily, the nature of proximal biological fluid, and the temperature [79]. Soft corona is a low affinity layer of proteins with a fast exchange over time. A recent model [80] suggests that hard corona is bound in a hard way to the NP surface and the soft corona is not directly bound to the NP but with a certain (low) degree of biomolecule interactions. As a result, the protein concentration, particle size, type of nanomaterial, and the surface properties are factors determining the layers of biomolecules and the protein corona density [81].

Depending on the type of administration routes, NPs are subjected to interactions with different kind of biomolecules [82]. The biological environment is another key factor that plays a determinant role in the protein corona formation: the media components, temperature, pH, and the physiological state of the medium. The “in vivo” protein corona formation of biomedical liposomes seems to be more complex than “in vitro” [83]. In consequence, the “in vivo” protein corona characterization is fundamental for biomedical applications.

Different methods and techniques are needed to determine proteins interactions in different biological media because of the large number of proteins at different concentrations that compete to functionalize with the NP surface [84]. Techniques usually described for protein corona evaluation are based on proteomic analysis [80], centrifugation, isothermal calorimetry titration, Ultraviolet and Visible (UV-Visible) spectrometry, Liquid Chromatography with tandem mass spectrometry (LC-MS/MS) quantification, and sodium dodecyl sulfate–polyacrylamide gel (SDS-PAGE) electrophoresis [85].

Therefore, it is essential to understand the relationship between the different properties of nanomaterials and a concrete biological environment in order to understand their stability, viability, behavior, and the results obtained in the different areas of research.

## 4. Applications of Nanoparticles

In this section, some of the NPs applications are briefly described in order to understand their broad potentials. Although we have focused on nanomedical applications, we should not forget all those other important non-biological applications that have improved the quality of human life (https://www.nanotechproject.org/inventories/) [86].

NPs have attracted great interest for nanobiotechnology applications (Table 1). The design of nanostructures controlling their surface properties is a strategy meant to achieve improved responses aimed at a medical application. Nano-biotechnology plays a central role in nanomedicine and other areas, which aspire to develop highly functional biosensors, molecular switches, and tissue analogs for organs of the body among others. The nano-biotechnological applications to disease treatment, diagnosis, monitoring by bioimaging, biosensing, and drug delivery have been referred to as nanomedicine. Nanomedicine holds significant potential to improve the efficacy of cancer immunotherapy.

### Nanomedical Applications: Immunotherapy

Immunotherapy has become one of the effective treatment modalities for cancer: cytokine therapy, checkpoint-blockade therapy, adoptive T-cell transfer, and Chimeric Antigen Receptor T(CAR-T) cell therapy [120]. Immunotherapy not only treats primary tumors but also prevents metastasis and recurrence. Another opportunity for combinatorial immunotherapy is based on NP platforms because of their improved methods for tumor-cell detection, tumor imaging, and their ability to efficiently deliver drugs to target sites and protect drugs from endogenous enzymes [121]. Therefore, it is relevant to highlight how NPs may be engineered to overcome immunotherapy obstacles. In this mini review, we have discussed how NPs properties affect a biological mechanism and how they influence cellular internalization, biodistribution, and elimination. Therefore, we have enough information to understand how they alter immune responses.

NPs can release agents in response to biochemical changes in the target micro-environment (pH, redox potential, and enzymes) or to external stimuli (light, electrical, and magnetic fields) [122]. Due to that, targeted delivery of NPs and controlled drug release may allow the activation of immunotherapies in the action sites [123]. The use of NPs for delivery antigens, adjuvants, and other therapeutic agents resulted in more specific targeting and a better outcome in contrast to conventional immunotherapy. Advanced biomaterials and drug delivery systems, such as NPs and the use of T cells, have been designed to improve immunotherapy [124]. Moreover, NPs can deliver cytotoxic agents to tumor cells killing most of all the target cells with low concentrations of immune-stimulating drugs thanks to their potential to amplify T cell responses [120].

NP physicochemical properties can be tuned to stimulate the innate immune cells and to promote NP-immune cell interactions, which is a good therapeutic option [125]. Different strategies to enhance the efficacy of NP immunotherapy are the following [126,127]: Controlling the hydrophobicity surface (using hydrophilic polymers such as PEG) and a shape and rigidity optimization of NPs must reduce nonspecific uptake, which results in an efficient internalization. Enhancing tissue and cell penetration has been possible using peptide and chemical modifications to the NP surface such as cyclic iRGD peptide (CRGDK/RGDP/EC). Another important factor is targeting NPs and their bio-distribution to immune cells with ligands on NPs, such as T lymphocyte or B lymphocyte targeting. Nano-sized NPs have the advantage of accumulating within the tumor microenvironment with specific targeting, which minimizes off-target toxicity [125]. Then, once NPs reach the target cell, their biological activity occurs when they travel to the suitable intracellular compartment [127]. As a result, cationic polymers, pH-sensitive biomaterials, virus-derived cell-penetrating peptides, and direct cytosolic delivery must be used on NP in order to conduct appropriate intracellular delivery of NPs. Lastly, another approach to control immunotherapy is controlling the release kinetic.

The most common nanocarriers allowing specificity are liposomes, micelles, dendrimers, gold NPs, iron oxide NPs, carbon NPs, and quantum dots (NPs for tumor immunotherapy). Liposomes [128] are highly biocompatible and can be functionalized. However, they are widely studied for cancer immunotherapy. Micelles have a range application in cancer treatment because of their biodegradability and nontoxicity formulations, which makes them suitable for carrying therapeutic payloads. In addition, dendrimers [129] offer a highly specific NP physical properties thanks to their stepwise branching synthesis. Inorganic nanoparticles are well studied, such as gold NPs [123]. AuNPs are bio-inert and non-toxic nanocarriers, which, depending on their size, charge, shape, and functional group, may contribute to the efficacy in accumulating different immune cells [125]. The most studied functionalization for cancer immunotherapy is nanoparticles based on poly(lactic-co-glycolic acid) (PLGA NPs) because of their acceptance and biodegradability. Rosalia et al. [130] studied PLGA NPs functionalized with a αCD40-monoclonal antibody agonistic vaccine targeting dendritic cells (DCs). Two different adjuvants targeting the toll like receptor (TLR) were encapsulated into PLGA NPs to induce potent CD8+ T cell responses. In vivo experiments in murine melanoma-OVA mouse model indicated that active targeting of DCs and vaccine delivery resulted in efficient priming of CD8+ T cells, tumor control, and prolonged survival of the tumor-bearing mice.

Several programs work in integrated and interconnected research focused on therapeutically modifying the tumour micro-environment, (re)activation of anti-cancer immunity, and corresponding Drug Delivery System (DDS) [131]. Initially, they aim to develop new tumor-targeted drugs to selectively block key innate and adaptive immune checkpoints, such as PD-1, TIM-3, and CD47, in the tumor micro-environment. Furthermore, they aim to develop new tumor-targeted drugs to selectively activate key co-stimulatory receptors of the tumor necrosis factor receptor superfamily (TNFRSF) in the tumor micro-environment.

On the other hand, suitable drug delivery systems (DDS) could be developed using modern drug formulations based on nanotechnology and surface chemistry to achieve tumor-localized release and optimal localized co-stimulation of anti-cancer immunity. These developments will be attended by label-free detection of protein interactions by means of advanced bioanalysis methods [132]. That could ensure induction and execution of anti-cancer immune responses in the absence of systemic immune-related side-effects.

Lastly, immunotherapies help to amplify the knowledge and manipulation of the immune system and nanotechnology may be the cause of engineering remarkable mechanisms to produce an effective and long-lasting immune response against cancer.

## 5. Conclusions and Perspectives

Nanotechnology has significantly impacted medicine. In the past decade, studies about the biological response to NPs are greatly investigated in parallel with nano-bio interactions, which have influenced NP design. Both were in concordance with the evolution of NPs for biomedical applications. Many studies have investigated and demonstrated that NPs can enter into the human system. Therefore, the NP characteristics on biological systems, such as their physicochemical properties (size, shape, surface, coating and morphology, surface charge, hydrophobicity, chemical composition, structure, and the state of agglomeration), the types of biomolecules present, and the bio-identity of NP protein corona are important issues to characterize in order to know how they interact with cells, organisms, biological medium, biomolecules, and other biological systems or even with other nanomaterials. These studies helped determine their possible biocompatibility and toxicity in biological micro-environments and to engineer nontoxic nanomaterials, which may be used in biomedical applications.

With the potentially wide application of NPs in the future, these may be extensively used in various fields, especially in immunotherapy for clinical diagnosis and therapy based on their size, biocompatibility, surface chemistry, and adjustable toxicity. Immunotherapy combined with nanomedicines may be used to treat different types of cancer due to their excellent efficacy in penetration, specific retention, and killing of tumor cells.

The human proteome study [133] can be an arduous and discouraging task due to the high number of proteins, encoded by around 25,000 different genes, from which multiple protein variants are generated by post-translational modifications. The concept of proteomics involves a comprehensive study on the structures, localizations, post-translational modifications, functions, and interactions of all proteins expressed by an organism at a certain time and under certain conditions. The nanotechnology field has been expanded by providing innovative methods capable of responding to proteomic demands. In this sense, nanotechnology applications in proteomics have established a novel technical platform termed “nanoproteomics.” Detection techniques without labels are useful in the study of protein interaction kinetics, thanks to avoiding steric impediments caused by the presence of labels. The design and development of new multi-functional platforms based on nanomedicine could be of great interest in the unlabeled detection of protein-protein interactions given the possibility of synthesizing de novo proteins “in vitro” in the presence of these nano-systems.

In conclusion, the progress in nano-bio studies can potentially improve nano-medical applications and ensure a sustainable future.

## Figures and Tables

**Table 1:** Main applications of nanoparticles in nanomedicine.

Applications	Findings	Conclusions	References
Tissue and implants engineering	Gold and titanium dioxide nanoparticles have been used to enhance cell proliferation rates for bone and cardiac tissue TiO_2_ nanoparticles conjugated with the polymer poly(lactic-co-glycolic acid) (PLGA), decrease harmful effects, match the nanostructured roughness of bone, and improve their cell performance. Nanofibers that serve as a peptide scaffold allow the regeneration of the axonal tissue.	Nanotechnology in tissue engineering is used to create, repair, and/or replace cells, tissues, and organs combining cells with bio-nanomaterials, and to provide the best micro-environment where cells must grow. Nano-scaffolds are used in tissue and implants engineering to regenerate central nervous system cells and possibly other organs.	[87,88,89]
Antimicrobial vehicules	Silver and titanium dioxide nanoparticles have antimicrobial properties that allow them to be used in surgical mask coatings by eliminating bacteria and viruses.	Drug coated nanoparticles have shown the potential to repel microorganisms and to act as a prevention tool. A unique property of nanomaterials is their high surface-to-volume ratio. Therefore, minuscule amounts of nanoparticles can lend substantial antimicrobial effects.	[90,91,92]
Gene delivery	Silica nanospheres functionalized with ammonium cation groups allow transfecting cell lipids, polymers, graphene, carbon nanotubes, nanospheres, and different types of inorganic particles to be used.	Nanoparticles have a great potential as vectors to deliver genetic material into living cells.	[93,94,95]
Cell separation	Magnetic nanoparticles (MNPs) allow magnetic bio-separations with low toxicity and high biocompatibility. At physiological pH and high salt concentrations, nanocomposites acquire a positive charge for easy electrostatic interactions. In general, the magnetic bio-separation of targeted biomolecules occurs thanks to the interaction between MNPs and a targeted molecule with a magnetic force.	Magnetic nanoparticles (MNPs) can be employed to separate biomolecules such as proteins, deoxyribonucleic acid (DNA), cells, bacteria, genes, and viruses depending on the specific functionalization of MNPs.	[95,96]
Biofuels	The use of Fe (0) nanoparticles favors the activity of bio-hydrogen production under anaerobic conditions.	Nanoparticles are attractive materials to produce sustainable energy resources, mainly biofuels, thanks to their large surface/volume ratio, which provides a greater number of active sites where they catalyze bio-hydrogen, biogas, biodiesel, and bioethanol production in a high yield.	[97,98,99]
Drug Delivery System (DDS)	A platinum derivate of a bile acid conjugated with multifunctional polymer-coated bio-ferrofluids as anti-tumor agent in osteosarcoma (MG-63) and T-cell leukemic (Jurkat) cells. The use of gold nanoparticles, polymer nanoparticles, or liposomes, among others, as excellent tumor peptide vaccine carriers play an important role in anti-tumor immunotherapy.	Nanoparticles-based drug delivery system (DDS) have been in the core of attention due to their unique and superior properties. These systems can enhance therapeutic efficacy by producing more favorable bio-availability, serum stability, and pharmacokinetics. Nanoparticle formulations provide better penetration and allow slow and controlled release of drug molecules at the target site for bioactivity	[100,101,102,103]
Anti-cancer chemotherapy	Chemical analogues with platinum (II)-based drugs or ruthenium-based antimetastatic agents have anti-cancer properties. The behavior and the biological properties of novel gold compounds containing different ligands have been reported for human ovarian cancer cells. One of the most studied gold (III) compounds is Auranofinan orally effective anti-rheumatic administered drug and an anti-cancer treatment.	Nanoparticles technology offers a series of advantages for drug delivery such as high loading yield, combination therapy, controlled release, prolonged circulation, and targeted delivery. Recently, platinum (II), ruthenium, and gold (III) compounds-based anti-cancer chemotherapy has been reported to kill cancer cells. Most of these studies have been done using proteomics approaches.	[49,104,105,106,107,108,109,110,111,112,113,114,115]
Biosensors	An enzyme-linked immunosorbent assay (ELISA) was developed in which nanoparticles (AuNPs) were used as carriers of the signalling antibody, anti-CA15-3-HRP, for the analysis of CA15-3, which is an important tumour marker useful for the follow-up of breast cancer. The use of magnetic nanoparticles as proximity sensors in magnetic resonance (NMR) is known as diagnostic magnetic resonance (DMR).	AuNPs can be used to improve the performance of studies, such as the classical ELISA test, which achieves greater sensitivities. The idea of using nanomaterials in biosensors arose from the possibility of lowering the detection limit (LOD) and improving the signal-to-noise ratio. A diagnostic magnetic resonance (DMR) is a powerful biosensor technology that offers advantages over other detection techniques as well as broad applicability for profiling different types of targets (DNA, proteins, metabolites, and cells).	[116,117,118,119]
